# A Rare Case of Cardiac Tamponade Induced by Chronic Rheumatoid Arthritis

**DOI:** 10.14740/jocmr2226w

**Published:** 2015-07-24

**Authors:** Tariq Yousuf, Jason Kramer, Adam Kopiec, Zachary Bulwa, Shuvani Sanyal, Jeffrey Ziffra

**Affiliations:** a Internal Medicine, Advocate Christ Medical Center, Oak Brook, IL, USA; b Rosalind Franklin University of Medicine and Science, North Chicago, IL, USA; c University of Illinois Chicago Medical School, Chicago, IL, USA

**Keywords:** Cardiac tamponade, Rheumatoid arthritis, Echocardiography, Pericardiocentesis, C-reactive protein

## Abstract

Rheumatoid arthritis (RA) is a chronic inflammatory autoimmune disease primarily involving the joint synovium. RA is a systemic disease which has many known extra-articular manifestations. We present a unique case of a patient with long standing RA who presented with a primary complaint of chest and back pain. Echocardiography revealed borderline normal left ventricular function and a large pericardial effusion with the finding of elevated intrapericardial pressure suspicious for cardiac tamponade. Infectious workup was all found to be negative. The presence and elevation of anti-cyclic citrullinated peptide antibody, rheumatoid factor and C-reactive protein (CRP) confirmed the patient was having an active flare-up of RA. It was determined that this flare-up was the cause of the cardiac tamponade. A pericardiocentesis was performed and 850 mL of bloody fluid was drained. The patient remained stable following the pericardiocentesis. At his follow-up visit, repeat echocardiogram showed no signs for pericardial effusion. Although there has been extensive study of RA, there are only a few documented cases noting the occurrence of cardiac tamponade in these patients. Therefore, it is important for the clinician to be aware of and recognize this potentially serious cardiac outcome associated with a common rheumatologic condition.

## Introduction

Rheumatoid arthritis (RA) is a chronic inflammatory autoimmune disease primarily involving the joint synovium. The severity of the disease and symptom manifestations are variable from patient to patient. As a systemic disease, it has many extra-articular symptoms. Cardiac involvement is common and includes rheumatic heart nodules, pericarditis, pericardial effusion and conduction abnormalities; most patients are often asymptomatic. Thirty to fifty percent of patients with RA have associated pericarditis but fewer than 10% are symptomatic [1, 2]. A majority of the times pericardial involvement is found incidentally on echocardiography or autopsy. Less than 10% of patients have clinically symptomatic pericarditis [3]. Restrictive pericarditis with cardiac tamponade is a rare complication and may present a diagnostic challenge [4]. Moreover, these patients have a poor prognosis. Progression to cardiac tamponade most often occurs in the context of active rheumatoid disease. Therefore, treatment and management should aim to control the inflammatory process of the disease. We present a case of cardiac tamponade secondary to active rheumatoid disease that was successfully treated by pericardiocentesis and colchicine.

## Case Report

**Figure 1:**
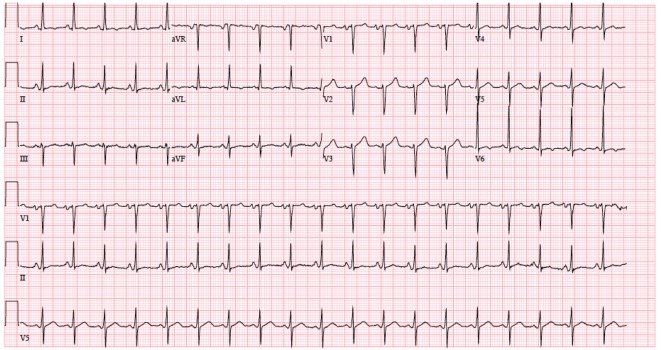
Initial EKG on January 21, 2015 at 9:04 am showing sinus tachycardia, left atrial enlargement and moderate voltage criteria for LVH.

**Figure 2:**
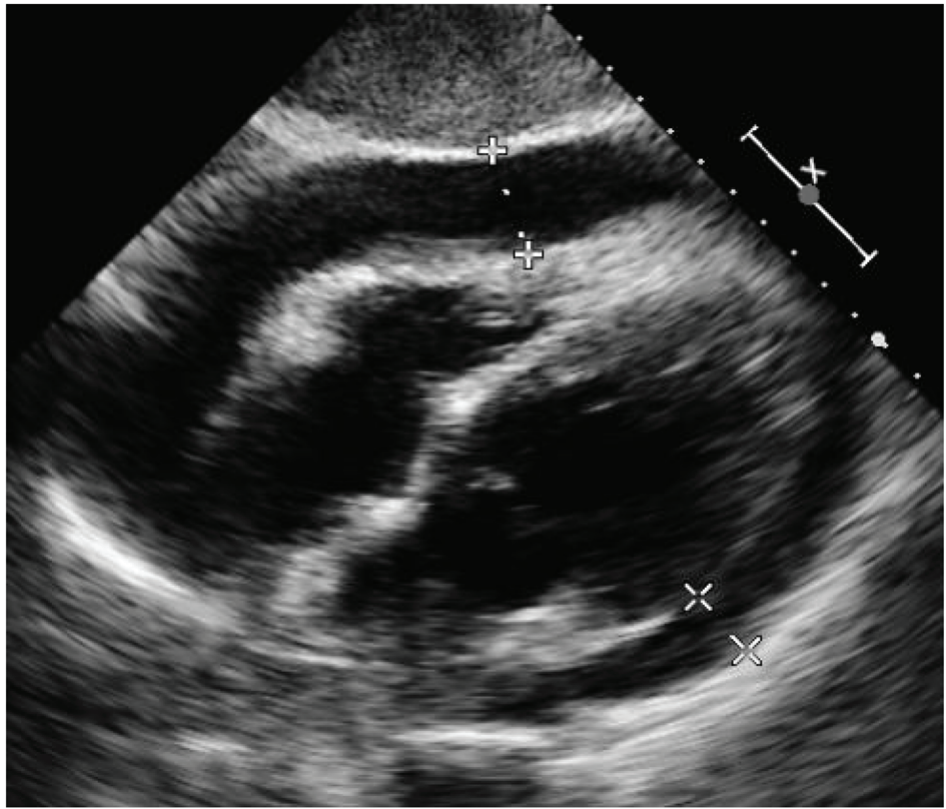
Initial echo on January 21, 2015 at 4:38 pm in subcostal view demonstrating large pericardial effusion, pericardial thickening and respiratory variations in the size of the left ventricle and inferior vena cava.

A 53-year-old Caucasian male was admitted to Advocate Christ Medical Center in January 2015 with multiple complaints including chest and back pain. The patient was diagnosed with RA back in 1989 and was initially treated with prednisolone and non-steroidal anti-inflammatory drugs (NSAIDs). Due to a recent history of perforated abdominal ulcer, his treatment regimen was modified. For the past 5 years, the patient has been on adalimumab and hydroxychloroquine for maintenance therapy of his RA. An electrocardiogram (ECG) (Fig. 1) and cardiac biomarkers were ordered and were found to be unremarkable. A chest X-ray was also performed which was remarkable only for cardiomegaly. In attempts to establish the etiology of the patient’s chest pain, an echocardiogram (echo) was ordered in the emergency department which showed evidence of a large pericardial effusion, pericardial thickening and respiratory variations in the size of the left ventricle and inferior vena cava (Fig. 2). Although the patient was initially stable and well appearing, his chest and back pain began to intensify as the workup was in progress. Therefore, the patient was transferred to the intensive care unit (ICU) to work up the etiology of the effusion. Physical examination in the ICU revealed a pulse of 101, respirations of 21, blood pressure of 120/95 and oxygen saturation of 97% on room air. Jugular venous distention (JVD) was appreciated and no friction rub was noted. Lungs were clear to auscultation and breath sounds were equal and symmetrical bilaterally.

Laboratory data included the following: white blood cell count (WBC) of 7.9 × 10^3^/mm^3^, hemoglobin concentration of 10.5 g/dL and platelet count 607 × 10^3^/mm^3^. The erythrocyte sedimentation rate (ESR) was elevated to 64 mm/h and the C- reactive protein was elevated to 4.4 g/dL. Rheumatoid factor (RF) was present with an elevated titer of 185 IU/mL and anti-citrullinated cyclic peptide (anti-CCP) was also positive at 122 units. Anti-nuclear antibody (ANA) was not detected. Titers for Epstein-Barr virus (EBV), cytomegalovirus (CMV), human immunodeficiency virus (HIV), and Coxsackie B virus were not detected.

Repeat ECG showed left atrial enlargement, moderate voltage criteria for left ventricular hypertrophy and non-specific T wave abnormality. Echo revealed borderline left ventricular function with large pericardial effusion and findings suggestive of elevated intrapericardial pressure indicating cardiac tamponade. Due to the escalation of his symptoms and the size of the tamponade, a pericardiocentesis was planned. Pericardiocentesis was performed and 850 mL of bloody fluid was removed. The findings from the pericardiocentesis are listed below (Table 1). LDH levels were not obtained.

**Table 1:** Pericardial Fluid Analysis

Pericardial fluid analysis	
pH	7.69
Glucose (mg/dL)	52
Total protein (g/dL)	5.9
Fluid volume (mL)	810
Nuc cell Ct (/μL)	1,465
Segs	81
Lymph	10
Monos	7
Eos	2
ANA titer	< 80
Rheumatoid factor (unit/mL)	185
C-reactive protein	4.4
CCP (unit)	122

A pigtail catheter was then placed for 2 days. The fluid showed no growth of both bacterial and fungal cultures and no acid fast bacilli were seen making mycobacterium tuberculosis highly unlikely. Furthermore, the fluid showed no evidence of malignancy. Computed tomography (CT) of the chest revealed evidence of pericardial thickening, so colchicine was administered by the recommendation of rheumatology. After the pericardiocentesis was performed, a repeat echo showed only a small amount of anterior pericardial effusion with normal left ventricular function and no evidence of tamponade.

Two days after the pericardiocentesis, the patient was transferred to the floors from the medical ICU in stable condition. A repeat echo showed a left ventricle normal in size with normal wall thickness and preserved systolic function as well as minimal evidence of pericardial effusion. The pigtail catheter was also pulled on this day with a minimal discharge of 75 mL over the previous 2 days. Repeat chest X-rays and echocardiograms were performed the next 2 days, which continued to show no significant evidence of pericardial effusion. The patient was discharged home on colchicine 0.3 mg every 6 h and told to continue his home medications of hydroxychloroquine 200 mg daily and adalimumab 40 mg daily. Two weeks after pericardiocentesis, the patient received follow-up chest X-rays and echocardiograms, both of which were unremarkable. The echo showed normal left ventricular systolic function with an ejection fraction of 50-55%, left ventricular wall thickness was normal and no effusion was seen.

## Discussion

Malignancy, collagen vascular disease, tuberculosis, hypothyroidism, various medications, viral, bacterial or fungal infection can cause pericardial effusions leading to cardiac tamponade. In this case, laboratory and pathology data showed no evidence of infection, thyroid dysfunction or malignancy. Moreover, the lab results demonstrated elevated ESR, CRP, RF and anti-CCP providing a strong indication of active rheumatic disease and a diagnosis of rheumatoid pericarditis.

Pericarditis in the context of RA occurs 30-50% of times and most often is only detected incidentally on echocardiogram or on post-mortem autopsy [1]. Symptomatic pericarditis occurs in less than 10% of these patients, and progression to cardiac tamponade is even rarer. According to the study done by Escalante et al in 1990, only five out of 960 patients with RA that were followed for over 11 years were found to have manifestation of cardiac compression [5]. Symptomatic pericarditis often takes place in association with other extra-articular manifestations. However in this case, significant other extra-articular signs of RA such as vasculitic changes, cutaneous ulcerations, scleritis or mononeuritis multiplex were not appreciated. The elevated inflammatory markers and RA-associated antibodies suggest that the pericardial effusion and subsequent tamponade occurred in this patient due to inflammatory and immunological processes.

Restrictive pericarditis and cardiac tamponade, resulting from RA-induced pericardial effusions, is a rare and life-threatening complication [6]. The rarity of this condition reflects in the paucity of literature documenting the effectiveness of different treatment approaches and the prognosis of these patients. For instance, there exists no evidence-based treatment regimen for rheumatoid pericarditis. According to existing reports, RA pericarditis has been treated successfully with NSAIDs, corticosteroids and immunosuppressive medication [7]. However, with rapidly progressing RA pericarditis resulting in massive pericardial effusion and subsequent tamponade, corticosteroids are inadequate and pericardiocentesis is warranted. Another case study of RA pericarditis-induced tamponade (by Imadachi et al) challenges practitioners to diagnose rheumatoid pericarditis clinically without biopsy [7]. Based on our findings, we draw the conclusion that surgical pericardial fluid drainage is indicated in circumstances of acute, life-threatening tamponade both as therapeutic and a diagnostic modality to confirm rheumatoid pericarditis. Simultaneous treatment of RA pericarditis with systemic corticosteroids and immunosuppressants are warranted due to the complicated sequelae.

Few studies have been conducted on RA as a cause for cardiac tamponade. As seen in our case, active RA has many extra-articular complications but clinicians must be cognizant of significant complications including cardiac tamponade. There are no clear guidelines on treatment regimen for RA-induced cardiac tamponade. Further investigations are needed to make proper treatment recommendations.
